# Kerion Celsi caused by *Microsporum gypseum* in a Chinese child, a case report

**DOI:** 10.1097/MD.0000000000028936

**Published:** 2022-04-01

**Authors:** Shuyue Wei, Haiying Wang, Ailan Li, Chunying Yuan

**Affiliations:** Department of Dermatology, Dongying People s Hospital, Dongying, Shandong, China.

**Keywords:** case report, guinea pig, kerion Celsi, *M gypseum*, tinea capitis

## Abstract

**Rationale:**

Kerion Celsi, a severe form of tinea capitis, is generally caused by zoophilic and geophilic fungi. This is the first report of an unusual case of kerion Celsi caused by *Microsporum gypseum* in a 6-year-old boy.

**Patient concerns:**

A 6-year-old boy presented to the dermatology clinic with the complaint of multiple pustules, edematous plaques over the scalp with hair loss for 1 month.

**Diagnosis:**

Clinical and laboratory investigations, including reverse transcriptase-quantitative polymerase chain reaction, confirmed M *gypseum* causing kerion Celsi.

**Interventions:**

Upon combination therapy using oral itraconazole and oral prednisolone along with the topical terbinafine, kerion Celsi remitted in the patient.

**Outcome:**

New hair growth was noted during the 4-month follow-up.

**Lesson:**

We presented the first case of kerion Celsi infection secondary to *M gypseum* that was probably transmitted from a guinea pig.

## 1 Introduction

Kerion Celsi, a local severe suppurative inflammation, is caused by a host cytotoxic reaction which is mediated by T cells rather than a bacterial infection. The onset and progression of tinea capitis are commonly attributed to zoophilic fungal infections caused by *Microsporum canis* and *Trichophyton tonsurans.*^[1–3]^ There is increasing evidence that the causative organisms related to tinea capitis differ depending on the geographical area. For example, *T tonsurans* has been primarily diagnosed in the United States of America, the United Kingdom, Canada, Brazil, and Japan. *T verrucosum* has been identified as a primary causal agent in Turkey. In China, *M canis* is a frequently diagnosed pathogenic agent in China.^[4]^ However, the disease is seldom caused by geophilic fungus, like *Microsporum gypseum* in China.^[5]^ Zhang et al^[5]^ reported a case of kerion Celsi infected by *M gypseum* in a boy undergoing dermatoplasty. Zhu et al^[6]^ retrospectively reviewed the incidence and pathogenesis of kerion Celsi for 19 years and found that *M gypseum* was responsible for infection in 4.21% cases. Herein, we firstly report a case of kerion Celsi caused by *M gypseum,* which was probably transmitted from guinea pigs.

## 2 Case presentation

A previously healthy 6-year-old girl (weight 30 kg) presented to the dermatology clinic with the complaint of multiple pustules, edematous plaques over the scalp with hair loss for 1 month. His parents reported a high-grade fever (<38.7°C) with an abscess formation for 4 days. Initially, he had been diagnosed with bacterial folliculitis by a general practitioner at a local hospital. He did not have any history of tinea manus and pedes or a family history of genetic diseases. There was no history of diabetes. He was administered topical and systemic antibiotics, including cefixime 50 mg/kg daily and mupirocin ointment twice a day for 4 days. However, his condition did not improve with the medications. Following this, he was brought to our center for dermatological evaluation. The patient confirmed having a guinea pig as a pet for 6 months.

**Figure 1:**
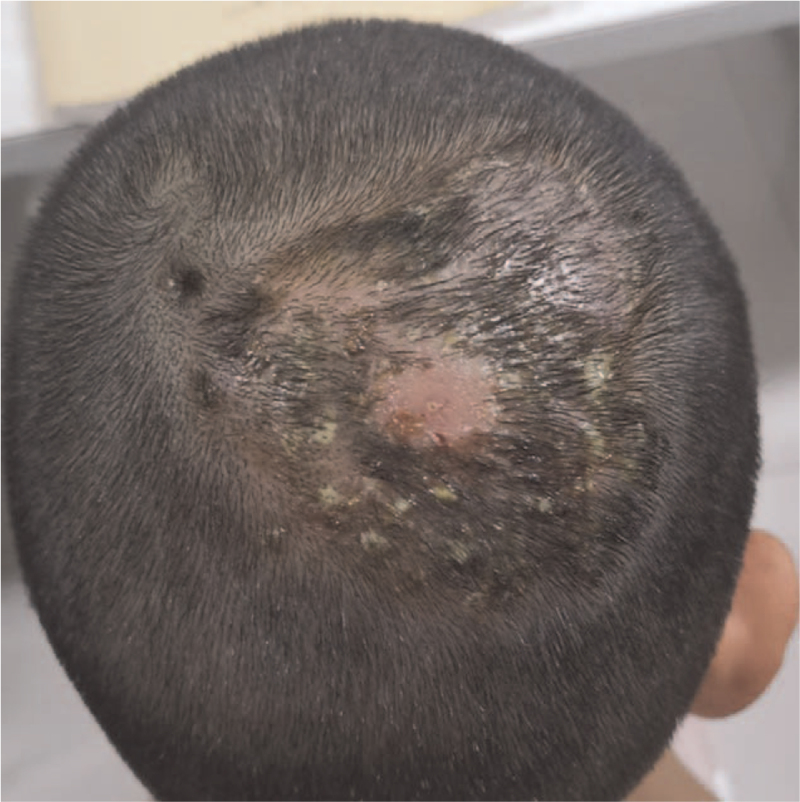
Clinical image of the crusty plaque along with purulent discharge. A round edematous plaque (7 × 8 cm) was visible in the occipital area of the scalp mixed with pustules, papules, purulent secretions, and diffuse alopecia.

On physical examination, a round edematous plaque (7 × 8 cm) was visible in the occipital area of the scalp mixed with pustules, papules, purulent secretions, and diffused alopecia (Fig. 1). On palpation, the lesion was soft and tender. The remainder of the physical examination was within the normal limit.

Blood investigations revealed neutrophilic leukocytosis white blood cells (white blood cell count: 18,930/μL [normal range 3500–9500], neutrophils: 13,100/μL [normal range 1800–6300], lymphocytes: 4260/μL [normal range 1100–3200], monocytes: 1300/μL [normal range 100–600], eosinophils: 60/ μL [normal range 20–520]) and an increased inflammation index [C-reactive protein]: 55.5 mg/L [normal range 0–10]). Gram staining and bacterial culture of the purulent secretions did not reveal any bacilli or bacterial infection.

**Figure 2:**
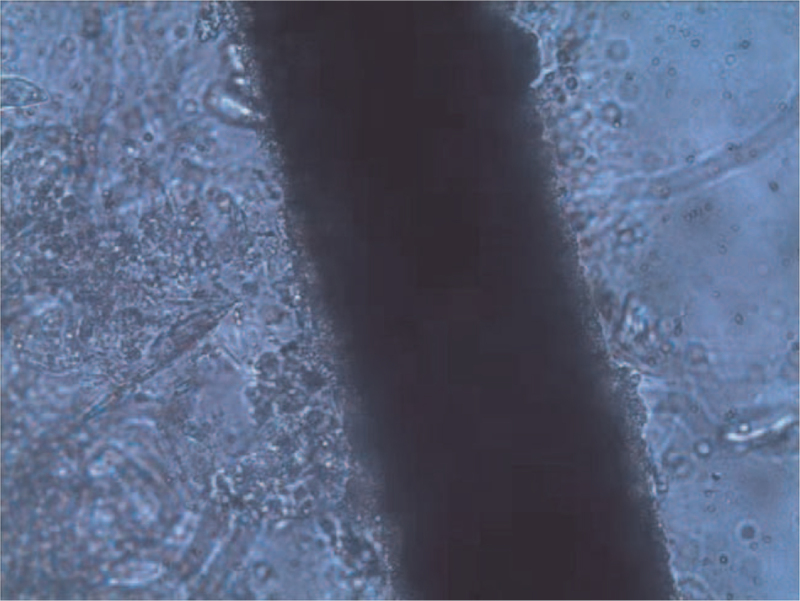
Exo-spores under fungal microscopic examination. A 10% potassium hydroxide examination of plucked hair and local skin demonstrated spores outside the hair.

**Figure 3:**
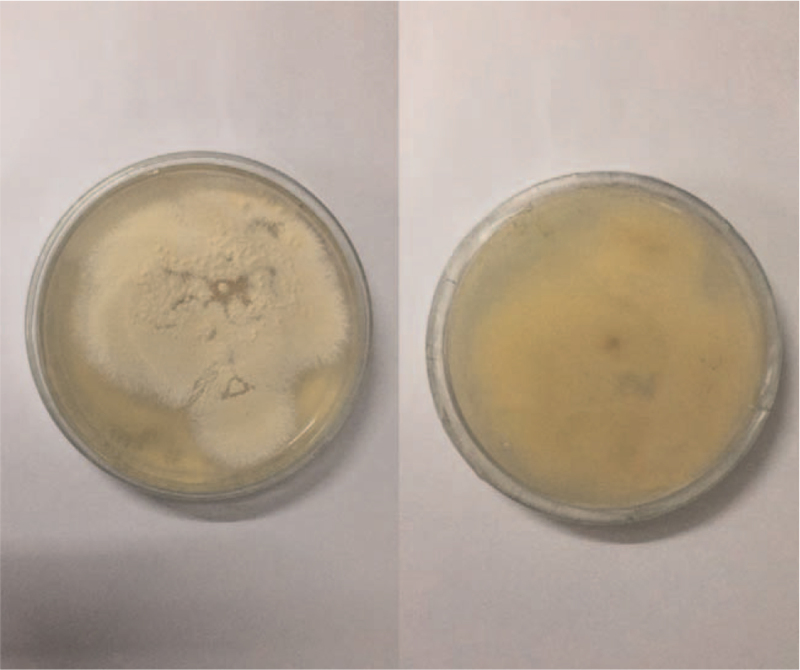
Culture on Sabouraud's glucose agar. Yellow-white powdery colonies with a powdery surface and a maize-yellow reverse at Sabouraud's glucose agar culture from the plucked hair follicles and purulent secretions at 25°C after 1 week produced.

**Figure 4:**
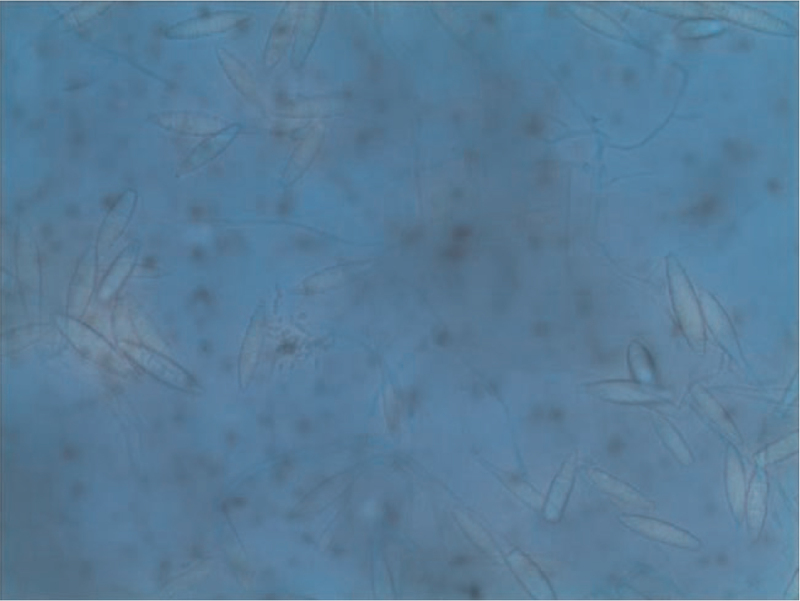
Microscopic examination of the morphology *M gypseum.* Fusiform macroconidia that were rather thin-walled, composed of 3 to 6 cells with rounded extremities divided into 4 to 6 cells, typical of.

A 10% potassium hydroxide examination of plucked hair and local skin demonstrated spores outside the hair (Fig. 2). Sabouraud's glucose agar culture at 25°C from the plucked hair follicles and purulent secretions after 1 week produced yellow-white powdery colonies with a powdery surface and a maize-yellow reverse (Fig. 3). The microscopic examination of the colony revealed fusiform macroconidia that were rather thin-walled, composed of 3 to 6 cells with rounded extremities divided into 4 to 6 cells, typical of M *gypseum* (Fig. 4).

The DNA of the fungus strain was extracted. The universal fungal primers ITS1 (5’-TCCGTAGGTGAACCTGCGG) and ITS4 (5’-TCCTCCGCTTATTGATATGC) were used for amplification by polymerase chain reaction (PCR).^[7]^ The amplification products were utilized for DNA purification and bidirectional sequencing. The sequences shared 99% homology with DNA sequences of *M gypseum* deposited in the GenBank.

Based on the history, clinical examination, and mycological and molecular findings, the patient was diagnosed with kerion Celsi caused by *M gypseum.*

The patient was administered itraconazole (oral, 150 mg/kg/ day–5 mg/kg/day) for 8 weeks and prednisolone (oral, 30 mg daily) sequentially for 2 weeks along with topical terbinafine ointment twice a day.

**Figure 5:**
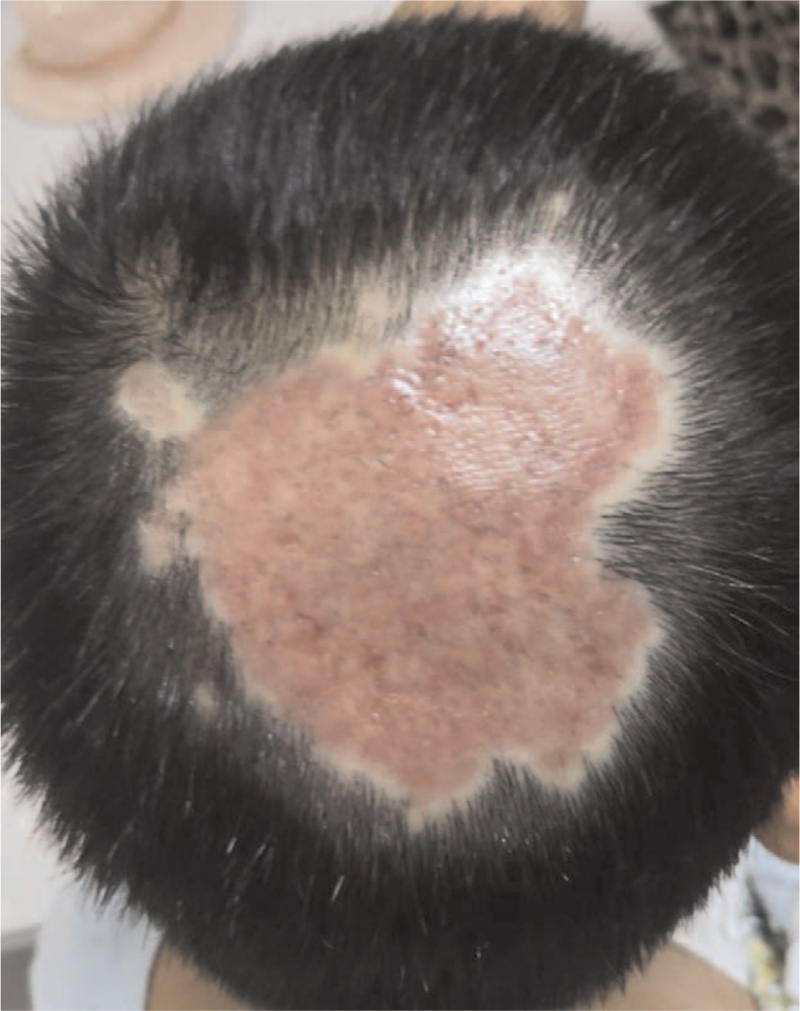
The disappearance of pustule after 2 months of treatment. After 2 months, the patient was cured without fungus detection on microscopic examination.

**Figure 6:**
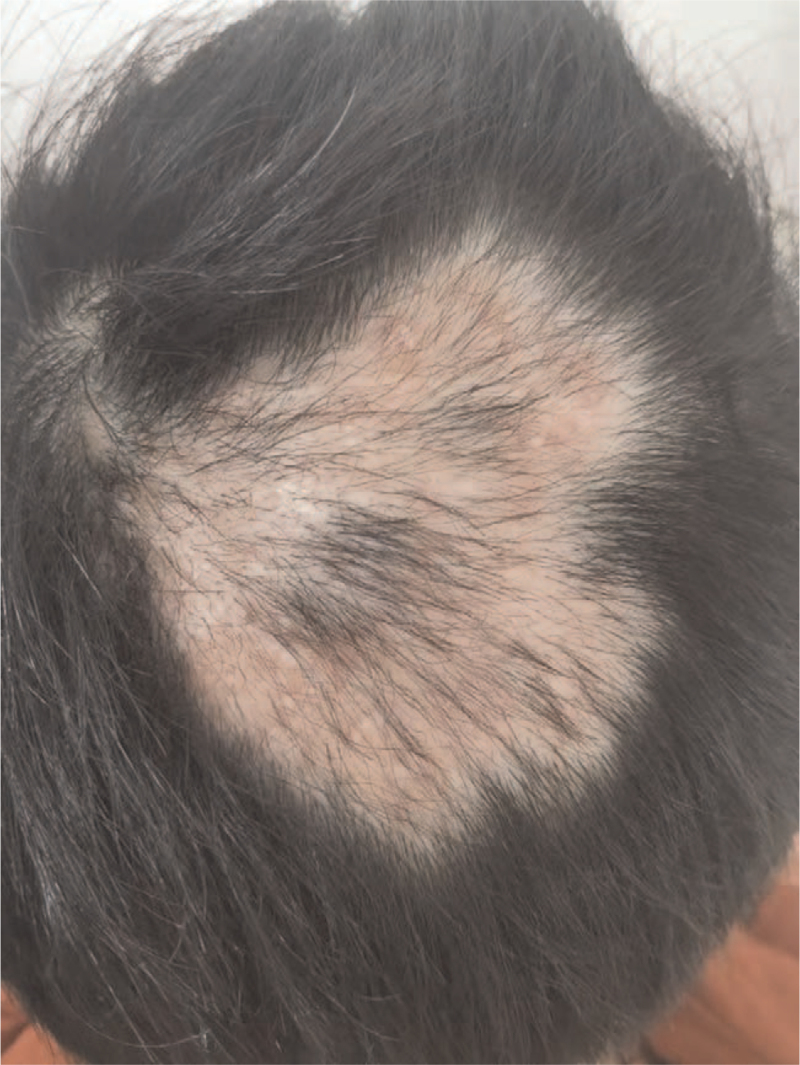
After 6 months of follow-up, the patients showing marked hair growth. No relapse was observed during the 4 months offollow-up, and partial hair growth in the patient was noted.

After 2 weeks of the treatment, the pain was significantly reduced, most of the pustules subsided, and the skin lesions were slightly higher than the skin. The color was dark red, and the fluctuation was not obvious. After 4 weeks of treatment, the abscess on the top of the head became flat with no purulent discharge, and the rash was dark red. The surface was dry. After 2 months of the treatment, the abscess on the top of the head subsided, leaving only alopecia spots (Fig. 5), and the mycoscopic examination was negative. There were no adverse reactions during the treatment, the liver and kidney functions were normal, and there was no recurrence during follow-up (Fig. 6).

The study was approved by the Ethics Committee of Dongying People's Hospital. Informed consent was obtained from the patient's parents.

## 3 Discussion

Kerion Celsi is an inflammatory and severe form of tinea capitis, characterized by boggy swelling, purulent discharge, alopecia, and lymphadenopathy.^[5,8]^ It is more commonly observed among children.^[2]^ It is typically caused by *Trichophyton* and *Micro-sporum* species.^[9,10]^ The dominant species observed in China was the zoophile *M canis* during 1986 to 2014.^[11]^ In contrast, kerion Celsi caused by *M gypseum* is less frequent. Only a few cases of tinea capitis caused by *M gypseum* have been reported in the Chinese population. *M gypseum* is a geophilic dermatophyte found in small mammals, such as cats and rodents, and can be transmitted to humans.^[12]^ A report has been published regarding the kerion Celsi secondary to *T mentagrophytes* species probably transmitted from rabbits.^[13]^ To the best of our knowledge, this is the first case of kerion Celsi infection secondary to *M gypseum,* probably transmitted from a guinea pig (according to the patient history); however, no etiological test was performed on the guinea pig.

As the kerion is an inflammatory disease with swelling and purulent discharge resembling a bacterial abscess, it can be easily misdiagnosed; thus, delaying the definitive treatment.^[14]^ In this case, the patient was timely presented to our hospital and underwent necessary laboratory investigations, and the diagnosis was confirmed using PCR.

The general practitioner initially administered oral and topical antibiotics without significant improvement. Moreover, some patients might be misdiagnosed and mismanaged and wrongly undergo surgical treatment, including incision and drainage, leading to delayed definitive treatment, unnecessary economic burden, risk of surgery, and even permanent scarring and alopecia negatively affecting the physical and mental health of patients.^[15]^

Since 1958, griseofulvin has been the first-line treatment for tinea capitis.^[10]^ Itraconazole has demonstrated comparable efficacy to griseofulvin and terbinafine,^[16,17]^ shortening the duration of therapy with fewer side effects.^[8]^ In addition, when the patient was treated using oral itraconazole with adjunction of oral prednisolone and the topical terbinafine. Consistently with the previous investigation, combination therapy was observed to be effective in reducing the inflammatory response.

In summary, clinicians should be highly suspicious of kerion Celsi when inflammatory lesions with purulent discharge are observed over the scalp, particularly in patients with ineffective antibiotic treatments or those in contact with pets. The microscopic examination and fungal culture or PCR should be performed to assist in correctly diagnosing the disease.

## Author contributions

Shuyue Wei and Haiying Wang were the patient's doctors, reviewed the literature and contributed to manuscript drafting; Ailan Li reviewed the literature and contributed to manuscript drafting; Chunying Yuan was responsible for the revision of the manuscript for important intellectual content; all authors issued final approval for the version to be submitted.

**Conceptualization:** Shuyue Wei.

**Data curation:** Shuyue Wei, Ailan Li.

**Methodology:** Haiying Wang.

**Visualization:** Chunying Yuan.

**Writing – original draft:** Shuyue Wei, Haiying Wang, Ailan Li, Chunying Yuan.
